# Nine Hole Peg Test and Transcranial Magnetic Stimulation: Useful to Evaluate Dexterity of the Hand and Disease Progression in Amyotrophic Lateral Sclerosis

**DOI:** 10.1155/2019/7397491

**Published:** 2019-11-07

**Authors:** David Czell, Christoph Neuwirth, Markus Weber, Sabine Sartoretti-Schefer, Andreas Gutzeit, Carolin Reischauer

**Affiliations:** ^1^Department of Neurology, Cantonal Hospital Winterthur, Winterthur, Switzerland; ^2^Department of Neurology, Spital Linth, Uznach, Switzerland; ^3^Neuromoscular Disease Unit/ALS Clinic, Cantonal Hospital St. Gallen, St. Gallen, Switzerland; ^4^Department of Radiology, Cantonal Hospital Winterthur, Winterthur, Switzerland; ^5^Department of Radiology, Paracelsus Medical University Salzburg, Salzburg, Austria; ^6^Department of Chemistry and Applied Biosciences, ETH Zürich, Zürich, Switzerland; ^7^Institute of Radiology and Nuclear Medicine, Clinical Research Unit, Hirslanden Hospital St. Anna, Lucerne, Switzerland; ^8^Department of Medicine, University of Fribourg, Fribourg, Switzerland

## Abstract

**Objective:**

Amyotrophic lateral sclerosis (ALS) is a neurodegenerative disease with involvement of the upper and lower motor neurons. Since the loss of fine motor skills is one of the earliest signs of ALS, the hypothesis was tested if the nine hole PEG test (NHPT) and transcranial magnet stimulation (TMS) with resting-motor threshold (RMT) could be useful in monitoring disease progression.

**Methods:**

We examined 28 ALS patients and 27 age-matched healthy controls. ALS patients and healthy controls underwent the nine hole peg test (NHPT) and TMS with RMT. Measurements in patients were repeated after three and six months.

**Results:**

At baseline, the median NHPT durations were 1,4-fold longer (*p* < 0.001), and TMS scores showed a significant 0.8-fold smaller score in ALS patients compared with healthy controls (*p* < 0.001). The comparison of three and six months versus baseline revealed significant differences for NHPT durations and ALSFRS-R in patients, whereas TMS scores did not significantly differ in the patients.

**Conclusion:**

NHPT seems to be a good tool to evaluate dexterity of the hand and the progression of the disease in ALS patients. TMS RMT to the hand muscles seems to be poorly qualified to evaluate the dexterity of the hand function and the course of the disease.

## 1. Introduction

Amyotrophic lateral sclerosis (ALS) is a neurodegenerative disease with an involvement of upper and lower motor neurons [1–3]. Consequently, patients can show atrophy and paresis of the affected muscles (lesion of the lower motor neurons (LMNs)) and a loss of dexterity of the hands and feet (lesion of the upper motor neurons (UMNs)). Involvement of the upper and lower motor neurons and patients with a primary involvement of the upper or lower motor neurons can vary [1–3]. The nine hole peg test (NHPT) was originally introduced by Kellor et al. in 1971 as a measure of dexterity [4]. In 1985, detailed test instructions and adult normative values according to hand, sex, and age were provided [5]. Since 1988, the test is used as an upper limb outcome measurement in multiple sclerosis, a disease with an involvement of the corticospinal tract [6]. In the last years, the NHPT could be assessed as a reliable test for dexterity in other diseases of the central nervous system such as stroke [7], traumatic brain injury [8], and cerebral palsy [9] but also for diseases with an involvement of the lower motor neurons as peripheral neuropathies [10, 11]. Transcranial magnetic stimulation (TMS) with resting-motor threshold (RMT) is a tool to show the involvement of the UMNs. In TMS studies, focal cortical changes and hyperexcitability as a specific feature of ALS [12–16] could be demonstrated. In the present study, NHPT and TMS with RMT are assessed in a cohort of ALS patients and healthy controls. Changes in these parameters over time and correlation with disease severity are analyzed.

## 2. Materials and Methods

### 2.1. Patients and Control Subjects

In this study, 28 ALS patients (mean age = 62.6 years, range = 48–84 years, 13 female, 15 male, 12 bulbar, 16 limb onset) were recruited. Four of them had definite, nine probable, ten probable laboratory-supported, and five possible ALS according to the revised El Escorial criteria [17]. Twenty-seven age-matched healthy controls (mean age = 63.5 years, range = 50–82 years, 13 female, 14 male) were recruited. They had no prior history of neurological disorders. All subjects (ALS patients and healthy controls) underwent the NHPT and TMS, and RMT was performed (see details below). The ALS patients and a subgroup of ten healthy subjects (mean age = 62.3 years, range = 55–72 years, five female, five male) underwent these measurements after three and six months, respectively. The ALS functional rating scale (ALSFRS-R, maximum score = 48) was used for all ALS patients to assess disease severity at each measurement time point with lower scores representing more severe disability [18].

### 2.2. Nine Hole Peg Test (NHPT)

The NHPT consists of a board (wood) with 9 holes (10 mm diameter and 15 mm depth) placed apart by 32 mm and a container for the pegs, a square box (100 × 100 × 10 mm) apart from the board. The participants were instructed to remove all the pegs from the holes, one by one, and place them into the container and then to take back the pegs from the container, one by one, and replace them into the holes on the board, as quickly as possible. The board should be placed at the client's midline, with the container holding the pegs oriented towards the hand being tested. Only the hand being evaluated should perform the test, and the hand not being evaluated is permitted to hold the edge of the board in order to provide stability. The scores are based on the time taken to complete the test activity, recorded in seconds. To this end, a stopwatch is utilized to record the time from the moment the participant touches the first peg until the moment the last peg enters the last hole.

### 2.3. Transcranical Magnetic Stimulation (TMS) with Resting-Motor Threshold (RMT)

The focal TMS of the cortical hand area of the left (L-M1) and right side (R-M1) was performed using a Magstim-200 stimulator. It has a figure-of-eight coil with external loop diameters of 90 mm (Magstim Company, Carmarthenshire, United Kingdom). It has also a monophasic current waveform. During the stimulation, the investigator performed the TMS tangentially to the skull. The handle was backward and laterally at a 45° angle to the sagittal plane. By producing the largest TMS amplitudes (stimulation of the hand areas elicited in the right and left abductor pollicis brevis, respectively), the optimal coil positions were found. By using the relative frequency method to the nearest 1% maximum stimulator output (MSO) [19], the resting motor threshold (RMT) was determined. We defined it as the minimum intensity which could elicit a motor evoked potential of >50 *μ*V peak-to-peak amplitude in at least 5 out of 10 subsequent trials. RMT is stated as percentage of MSO.

### 2.4. Statistical Analysis

Statistical analysis of the data was performed in R (a language and environment for statistical computing, R Foundation for Statistical Computing, Vienna, Austria. 2018). NHPT durations and TMS thresholds were log-transformed before regression analysis, while ALSFRS-R was analyzed on a normal scale as verified by preliminary analysis of quantile comparison plots. Differences between ALS patients and healthy controls were analyzed by linear regression models. Changes over time were evaluated using linear mixed-effect models with baseline values as reference. All regression models were adjusted for age and gender. The estimates of the effect sizes derived from the regressions were difference of mean values for ALSFRS and back-transformed geometric mean ratios for NHPT and TMS thresholds. In addition, the corresponding 95% confidence interval as well as the *p* value was indicated.

Correlations of NHPT duration and TMS scores, respectively, with disease severity as assessed by the ALSFRS-R were evaluated using Spearman's rank correlation.

## 3. Results

Of the initial 28 ALS patients, eight were lost to follow-up: two patients deceased after the baseline measurement, five patients could not further participate in the study after a rapid deterioration of their respiratory function, and one patient withdrew willingness to participate in the study after the initial measurement. Thus, longitudinal analysis of the parameters was performed across 20 ALS patients (mean age = 62.5 years, range = 48–84 years, 10 female, 10 male).

### 3.1. Patients and Controls

At the baseline, NHPT durations were 1,4-fold longer (95% CI: 1.2, 1.7) in ALS patients compared to healthy controls (*p* < 0.001, see Table 1). TMS scores also significantly differed between ALS patients and healthy controls: ALS patients showed a 0.8-fold smaller score (95% CI: 0.65, 0.98, *p*=0.031) than healthy patients. In Table 2, median (range) values are indicated for NHPT duration and TMS scores, while mean ± SD are indicated for ALSFRS-R over time. The comparison of three and six months versus baseline by mixed-effect models adjusted for age and gender revealed significant differences for NHPT durations and ALSFRS-R in patients, whereas TMS scores did not significantly differ. In the healthy controls, no significant changes were detected neither in the NHPT durations nor the TMS scores over time. Comparing the relative effect sizes of NHPT durations and ALSFRS-R scores in patients, NHPT showed a stronger effect size than ALSFRS-R scores, while NHPT values were 15% and 16% larger after three and six months compared with baseline, respectively, ALSFRS-R values were only about 5% and 10% lower at the same time.

There was no significant correlation between ALSFRS-R and NHPT durations in the ALS patients, as assessed by Spearman's rank correlation (*r* = −0.05, *p*=0.66).

There was no significant correlation between ALSFRS-R and TMS scores in the ALS patients, as assessed by Spearman's rank correlation (*r* = 0.08, *p*=0.55).

There was a highly significant moderate negative correlation between the NHPT durations and the ALFRS-R: handwriting scores (*r* = −0.53, *p* < 0.001).

There was no significant correlation between the TMS scores and the ALFRS-R: handwriting scores (*r* = 0.13, *p*=0.31).

## 4. Discussion

In this study, the NHPT durations and the TMS with RMT scores in the disease progression of patients with ALS were investigated. TMS with RMT studies were performed earlier with ALS patients [20, 21]. NHPT were only investigated in an exploratory study with only one measurement and not over time like in this study [22]. Also, both measurements (TMS RMT and NHPT) were not combined before in a study. NHPT duration is a reliable tool that has been used in studies investigating diseases affecting UMNs and LMNs for years [6–11]. In this study, significantly longer NHPT durations were observed in patients compared with controls. This most likely mirrors the fact that in many patients, impairment of dexterity is an early feature or will develop during the course of the disease. This is supported by a significant change of NHPT durations in the ALS patient over time. This suggests that the NHPT is a suitable tool to evaluate dexterity and progression in ALS patients. This is in line with previous studies of other diseases with involvement of upper or lower motor neurons that indicate that the NHPT is a suitable tool for patients with a primary involvement of the UMNs and LMNs [6–11]. In our study, we found a significant difference between ALS patients compared with controls using TMS. This result is in line in with previous studies in which hyperexcitability could be detected in ALS patients [12, 13, 20, 21, 23]. The development of hyperexcitability in ALS is a well-known phenomenon and develops during the early stages of the disease. In this study, the cortical threshold, which is lower in ALS patients than in controls, is the sign for hyperexcitability. It was reported to be lowest early in the course of ALS and to subsequently increase as the disease progresses [12, 20, 21, 23]. No significant changes of the TMS RMT scores over time were observed in the present work. Maybe, the progression of deterioration of the pyramidal tract in our patients was too slow. Perhaps, this could be investigated in further studies. But our results are in line with earlier findings that RMT TMS is not as sensitive and specific as threshold tracking TMS in ALS [13, 24].

## 5. Conclusion

NHPT seems to be a good tool to evaluate the dexterity of the hand and the progression of the disease in ALS patients. TMS with RMT to hand muscles seems to be poorly qualified to objectively document the course of the disease and the deterioration of the hand function.

## Figures and Tables

**Table 1:** Mean values as well as standard deviations and median values as well as range, respectively, of the parameters for patients and controls at baseline. Log-transformed NHPT und TMS values were analysed by linear regression adjusted for age and gender. Geometric mean ratios (GMR) and 95% CI and *p* value are indicated. Statistically significant values are designated by an asterisk.

Parameter	Mean ± standard deviation/median (range)		
	Patients	Controls	GMR (95% CI)
NHPT	24 s (16–75 s)	19 s (15–28 s)	1.41 (1.20, 1.66)
TMS	42% (34.5–46.5%)	52% (47–60%)	0.80 (0.65, 0.98)
ALSFRS	40.2 ± 3.1	n.a.	n.a.

n.a.: not applicable.

**Table 2:** Mean values as well as standard deviations and median values as well as range, respectively, of the parameters for each group at baseline as well as at three and six months.

Parameter	Group	Mean ± standard deviation/median (range)		
		Baseline	3 months	6 months
NHPT	Patients	25 s (17–75 s)	28 s (18–130 s)	31 s (17–56 s)
	Controls	19.1 ± 2.8 s	20.0 ± 3.9 s	20.3 ± 2.7 s
TMS	Patients	39.4% ± 11.0%	38.9% ± 10.5%	37.9% ± 8.5%
	Controls	52.1% ± 9.9%	52.2% ± 8.4%	53.2% ± 8.9%
ALSFRS-R	Patients	39.8 ± 3.3	38.2 ± 3.8	36.5 ± 4.1
	Controls	n.a.	n.a.	n.a.

n.a.: not applicable.

## Data Availability

The data used to support the findings of this study are available from the corresponding author upon request.

## Abbreviations

ALS: Amyotrophic lateral sclerosis
ALSFRS-R: Amyotrophic lateral sclerosis functional rating scale revised
APB: Abductor pollicis brevis muscle
MSO: Maximum stimulator output
NHPT: Nine hole PEG test
RMT: Resting-motor threshold
TMS: Transcranial magnet stimulation.
